# Transcriptomic and lipidomic profiling provide novel insight into the pathogenesis of monogenic *SGMS2*-related osteoporosis

**DOI:** 10.1093/jbmrpl/ziaf128

**Published:** 2025-08-13

**Authors:** Sandra Pihlström, Ali Oghabian, Kirsi Määttä, Jelmer Legebeke, Riikka E Mäkitie, Philippe M Campeau, Paulien A Terhal, Lorenzo D Botto, Vesa M Olkkonen, Outi Mäkitie, Minna Pekkinen

**Affiliations:** Folkhälsan Research Center, Institute of Genetics, 00250 Helsinki, Finland; Research Program for Clinical and Molecular Metabolism, Faculty of Medicine, University of Helsinki, 00014 Helsinki, Finland; Folkhälsan Research Center, Institute of Genetics, 00250 Helsinki, Finland; Research Program for Clinical and Molecular Metabolism, Faculty of Medicine, University of Helsinki, 00014 Helsinki, Finland; Folkhälsan Research Center, Institute of Genetics, 00250 Helsinki, Finland; Research Program for Clinical and Molecular Metabolism, Faculty of Medicine, University of Helsinki, 00014 Helsinki, Finland; Department of Molecular Medicine and Surgery and Center for Molecular Medicine, Karolinska Institutet, 171 777 Stockholm, Sweden; Folkhälsan Research Center, Institute of Genetics, 00250 Helsinki, Finland; Research Program for Clinical and Molecular Metabolism, Faculty of Medicine, University of Helsinki, 00014 Helsinki, Finland; Department of Otorhinolaryngology—Head and Neck Surgery, Helsinki University Hospital and University of Helsinki, 00014 Helsinki, Finland; Medical Genetics Division, Department of Pediatrics, CHU Sainte-Justine, Montreal, QC H3T 1C5, Canada; Department of Genetics, Utrecht University Medical Center, 3508 TC Utrecht, The Netherlands; Division of Medical Genetics, Department of Pediatrics, University of Utah, Salt Lake City, UT 84112, United States; Minerva Foundation Institute for Medical Research, 00290 Helsinki, Finland; Department of Anatomy, Faculty of Medicine, University of Helsinki, 00014 Helsinki, Finland; Folkhälsan Research Center, Institute of Genetics, 00250 Helsinki, Finland; Research Program for Clinical and Molecular Metabolism, Faculty of Medicine, University of Helsinki, 00014 Helsinki, Finland; Department of Molecular Medicine and Surgery and Center for Molecular Medicine, Karolinska Institutet, 171 777 Stockholm, Sweden; Children’s Hospital, University of Helsinki and Helsinki University Hospital, 00014 Helsinki, Finland; Folkhälsan Research Center, Institute of Genetics, 00250 Helsinki, Finland; Research Program for Clinical and Molecular Metabolism, Faculty of Medicine, University of Helsinki, 00014 Helsinki, Finland; Children’s Hospital, University of Helsinki and Helsinki University Hospital, 00014 Helsinki, Finland

**Keywords:** calvarial doughnut lesions with bone fragility, sphingomyelin synthase 2, sphingomyelin metabolism, RNA sequencing, lipidomic

## Abstract

Heterozygous pathogenic variants in the *SGMS2* gene, encoding the sphingomyelin-synthesizing enzyme sphingomyelin synthase 2, cause a rare monogenic form of osteoporosis with low bone density, fractures, bone deformities, sclerotic cranial lesions, and occasionally, neurological symptoms. Three disease-causing heterozygous *SGMS2* variants have been reported: c.148C>T (p.Arg50*), c.185T>G (p.Ile62Ser), and c.191T>G (p.Met64Arg). This study examined the cellular mechanisms of *SGMS2*-related osteoporosis and skeletal dysplasia through transcriptomic and lipidomic profiling of serum and fibroblasts from patients and controls. Bulk RNA sequencing and SCIEX lipidyzer-based lipidomics were employed. Differential expression analysis revealed 215 upregulated and 58 downregulated genes enriched in 169 Gene Ontology Biological Processes related to skeletal, neurological, ocular, muscular, and membrane functions. Pathway analysis revealed enriched pathways associated with interleukin signaling, electrical transmission across gap junctions, and circadian clock. Four lipid metabolism pathways were enriched: PPARα regulation, glycerophospholipid biosynthesis, phospholipid metabolism, and lipid metabolism. Lipidome analysis failed to detect significant differences between fibroblasts of patients and controls, while revealing 55 upregulated lipids, predominantly triacylglycerols (TAGs), but no downregulated lipids in serum of the patients. These findings suggest that *SGMS2* variants modulate circadian rhythm and gap junction assembly, adversely affecting bone health and homeostasis, and may affect neuron-supporting cells in *SGMS2*-related osteoporosis.

## Graphical Abstract

**Graphical Abstract:**
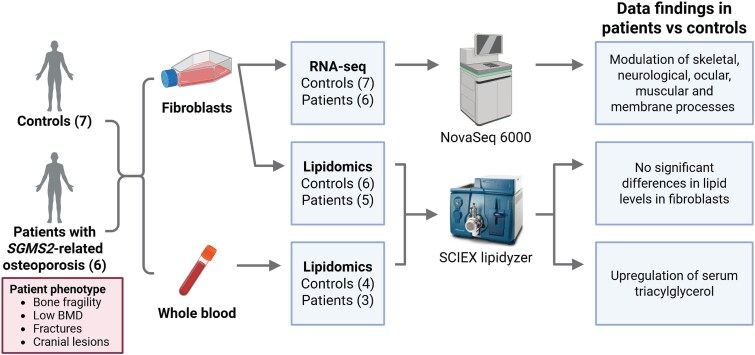

## Introduction

Calvarial doughnut lesions with bone fragility (CDL) with or without spondylometaphyseal dysplasia (OMIM #126550) is a rare autosomal dominant bone disease caused by pathological variants in *SGMS2*, the gene encoding sphingomyelin synthase 2 (SMS2).^1^^,^^2^ The disease either manifests as early-onset osteoporosis or as a more severe infancy-onset skeletal dysplasia, which are both characterized by a variable degree of bone fragility with low BMD, spinal and long-bone fractures, and multiple sclerotic cranial lesions. In addition to skeletal characteristics, neurological symptoms, such as facial nerve and ocular palsies, migraine, and diverse paresis may occur.^2–5^ Furthermore, CDL has been associated with myopia, glaucoma, and muscle function deficits.^2^^,^^3^^,^^5^ The clinical presentation of CDL varies greatly depending on the underlying *SGMS2* variant.^5^ To date, 3 heterozygous disease-causing *SGMS2* variants have been identified: c148C>T (p.Arg50*) leading to early-onset osteoporosis, c.185T>G (p.Ile62Ser) and c.191T>G (p.Met64Arg) leading to a more severe spondylometaphyseal dysplasia.^2^

Histomorphometric evaluation of patients’ bone biopsies showed an overall decrease in bone volume, a disorganized collagenous network, reduced mineral content, and increased heterogeneity in matrix mineralization.^2^ Although these findings suggest increased osteoclast numbers, functional analysis found no differences in osteoclast morphology or resorptive capacity.^2^ Further evaluation of the changes in bone mineral and dynamic properties revealed severe material defects specifically in bone matrix mineralization, osteocyte orientation and the osteocyte canalicular network.^6^ In vitro studies have revealed *Sgms2* expression in cultured murine osteoblasts, bone marrow macrophages, and osteoclasts.^2^ In addition, *SGMS2* transcripts are found in human skin fibroblasts and various other human tissues, including brain, heart, kidney, liver, muscle, and stomach.^7^

Sphingomyelin (SM), the most prevalent sphingolipid (SL) and a key element of animal cell membranes, is produced through a process catalyzed by SMS2. SMS2 promotes the transfer of phosphocholine from phosphatidylcholine onto ceramide (CER) leading to the generation of SM while also producing diacylglycerol (DAG) as a byproduct.^7^ Normally, SMS2 is produced in the endoplasmic reticulum (ER) before being transported to the plasma membrane, where SM is synthesized. However, *SGMS2* variants affect the cellular localization of SMS2 resulting in misdirected SM synthesis and disruption of plasma membrane SM asymmetry. The missense variants uphold SMS2 in the ER, while the p.Arg50* variant dislocates SMS2 into the *cis*/medial-Golgi.^8^

Sphingolipid metabolism plays a critical role in skeletal and neural homeostasis;^5^ however, the underlying molecular mechanisms remain elusive and the role of SL metabolism in muscle and eye physiology remains unclear. Thus, it is imperative to investigate the effects of *SGMS2* variants on gene transcription and lipid composition to understand how the pathogenic variants lead to clinical manifestations. In the present study, we have evaluated fibroblast transcriptomic and both fibroblast and serum lipidomic profiles in patients with CDL and healthy controls by utilizing bulk RNA sequencing (RNA-seq) and SCIEX lipidyzer-based lipidomics analysis.

## Materials and methods

### Study authorization, participants, and sample collection

The study was authorized by the Helsinki University Hospital’s Ethical Committee; research permit: HUS/265/2023, ethical permission: HUS/404/2018. All study participants or their guardians have signed an informed consent. Personal information and all omics data have been processed in accordance with EU’s General Data Protection Regulation (GDPR). Skin biopsies, utilized in RNA-seq and lipidomics analysis, and blood samples, utilized in lipidomics analysis, were collected from 6 individuals harboring a *SGMS2* pathogenic variant and from 7 healthy sex- and age-matched controls. All affected patients were clinically diagnosed with CDL and were verified to carry one of the known heterozygote pathogenic variants in *SGMS2*: p.Arg50*, p.Ile62Ser, or p.Met64Arg, identified through whole-exome sequencing and confirmed by Sanger sequencing, as described.^2^^,^^4^ The patients’ clinical and radiographic data were collected from hospital records.

### Sex as a biological variable

Our study investigated both male and female individuals with heterogeneous pathogenic *SGMS2* variants, along with healthy age- and gender-matched controls, and similar findings are reported for both sexes.

### Isolation and culture of primary fibroblasts

Skin biopsies from affected patients and healthy controls were processed into primary dermal fibroblasts as previously described.^9^ The cells were cultured in Dulbecco’s Modified Eagle’s Medium (DMEM) (12492013, Gibco) containing 10% fetal bovine serum (FBS) (S-FEB-SA-015, Serana), 100 IU/mL penicillin, 100 μg streptomycin (15140122, Gibco), and 2 mM glutamax (35050-038, Gibco). The cell culture was maintained in a water-saturated atmosphere with 5% CO_2_ and 95% air at 37 °C in the incubators. Each fibroblast line was seeded into a Nunc EasYFlask Cell Culture Flask (156499, Thermo Scientific). When confluent, the cells were collected into a pellet for future procedures.

### RNA isolation for bulk RNA-seq and qRT-PCR

Total RNA was isolated from fibroblasts using the RNeasy mini kit (74104, Qiagen). The extracted RNA was DNase treated by utilizing the DNA-free DNA Removal Kit (AM 1906, Invitrogen). Each procedure was completed according to manufacturer’s protocol. RNA concentration and RNA integrity were confirmed by RNA quality control (QC) analyses (TapeStation 42000 analysis and Qubit analysis), provided by the Biomedicum Functional Genomics Unit (FuGU) at the Helsinki Institute of Life Science and Biocenter Finland at the University of Helsinki. All RNA samples obtained an RNA integrity number (RIN) higher than 9.7.

### RNA sequencing

Following RNA isolation, RNA-seq libraries were generated using the NEBNext Ultra II Directional RNA Library Prep kit (E7770L, New England Biolabs), according to manufacturer’s protocol. Library QC was performed using MiSeq V2 Nano (Illumina) and library quantification was performed by qPCR, using Collibri Library Quantification kit (A38524100, Invitrogen) and QuantStudio 5 instrument (A28574, Applied Biosystems), before committing to a full-scale sequencing. Sequencing was performed on the Custom Novaseq S1 platform using 2x150 bp paired-end reads for analysis (Illumina). The RNA-seq service was provided by the FuGU at the Helsinki Institute of Life Science and Biocenter Finland at the University of Helsinki. RNA-seq results were verified by qRT-PCR.

### RNA sequencing data analysis

Sequenced RNAs from fibroblasts extracted from 6 affected patients and 7 healthy control individuals were analyzed. The quality of the sequence reads was assessed using FastQC and MultiQC.^10^^,^^11^ Illumina-specific sequences (such as the adapter sequence) and sequences with low quality were trimmed from the sequence reads.^12^ Threshold of Quality >3 was used for the sequences at beginning and end reads. The minimum read length cutoff of 36 nucleotides was considered. The size of sliding window was set to 4 and the minimum threshold for the average quality was set to 15. Even though rRNAs depletion was used at the library preparation stage, we filtered the reads that still mapped to the rRNAs using the SortMeRNA software to prevent any possible effects by the RNA read count values in the downstream analysis.^13^ The sequence reads were mapped to the Human genome (GRCh38.p13, GENCODE v41) using the splice-aware alignment software STAR (V2.7.10a) (Supplement).^14^ Subsequently, the mapped reads were summarized using HTSeq (with default parameter settings) to estimate how many reads are sequenced from each transcript.^15^ Comparing the patient samples to the controls, the significantly differentially-expressed genes were detected using DESeq2 (by applying an FDR < 0.05 cutoff).^16^ The analysis was adjusted for biases introduced by the different sex- and age-groups of the studied individuals by defining these factors as covariates in the design model. Furthermore, we used ASHR (ie, supported by DESeq2) to achieve expression log-fold-change values that are not biased by the read-counts levels of the genes.^17^ The Gene Ontology (GO) Biological Process categories enriched in the significantly differentially-expressed genes were extracted using topGO.^18^ Finally, for a simpler interpretation of the Enriched GOs, we used the simplifyEnrichment R/Bioconductor package to group the GO categories based on their semantic similarities.^19^ To carry out pathway analysis for the significant differently expressed genes, we used Reactome.^20^ We used the Benjamini–Hochberg method to adjust for multiple testing throughout our analyses.^21^ Four affected patients were heterozygous for the p.Arg50* variant. One was heterozygous for the p.Ile62Ser variant, and one for the p.Met64Ser variant. We carried out all the analyses twice. First, we compared all the patients to the controls. Second, we compared the patients with the p.Arg50* variant to the controls. In the end, significantly up- and downregulated genes (when comparing patient samples to controls), together with their associated biological processes were discovered, while preventing the possible effects that may have been caused by poor quality sequence reads, varied sex and age of the studied individuals, or multiple testing. The RNA splicing events present in the patients and controls were compared, using the STAR aligned BAM files and the Integrative Genomics Viewer v2.16.2, to assess whether the variants p.Arg50*, p.Ile62Ser, or p.Met64Ser causes aberrant alternative splicing within the *SGMS2* gene.

### cDNA synthesis and qRT-PCR

Quantitative reverse transcription PCR was used to validate RNA-seq results by determining the relative mRNA expression levels for *ELFN1*, *MOXD1*, *CUL7*, *ACTC1*, *JCAD*, and *NACC2* (normalized to *TBP*). Isolated RNA was reverse-transcribed into complementary DNA (cDNA) using QuantiTect Reverse Transcription Kit (205311, Qiagen). Gene expression analyses were performed by qRT-PCR, using PowerUp SYBR Green Master Mix (A25742, Applied Biosystems) and the CFX96 Touch Real-Time PCR Detection System (1 845 097, BioRad). The cycling parameters were the following: 50 °C for 2 min, 95 °C for 2 min, 40 cycles of 95 °C for 15 s, and 60 °C for 60 s. At the end of the cycles, a melting curve analysis was performed to ensure that only a single amplicon was acquired. Primers used for target genes and the reference gene are included in the supplement. The results were evaluated using Bio-Rad CFX Maestro Software (Bio-Rad Laboratories). The relative gene expression was quantified using the ΔΔCt method.^22^

### Sample preparation and lipidomics analysis

We drew 10 mL whole blood from 3 individuals with a p.Arg50* variant and 4 gender- and age-matched controls. The blood was left in room temperature for 30-60 min, to allow the blood to clot. The clot was removed by centrifuging at 2500 × *g* for 10 min. The remaining supernatant, approximately 4-5 mL, is the designated serum, which was stored in −80 °C, before proceeding to lipidomics analysis.

Fibroblasts used for lipidomic analysis were seeded to achieve a final concentration of 1 million cells/sample. Once the desired cell count was reached, the growth medium was aspirated. The cells were washed twice with PBS and trypsinized for 3-5 min at 37 °C. The cells were centrifuged at 339 × g for 5 min at 4 °C, and the supernatant was removed. Next, cells were washed twice in 2× volume of deionized water, without disturbing the cell pellet, before the water was aspirated, and the cell pellet frozen with liquid nitrogen. The cell pellet was stored at −80 °C before proceeding to lipidomics analysis.

Extraction of lipids from frozen blood serum and frozen cell pellets was performed using the liquid–liquid extraction (LLE) method with ethyl acetate and methanol as the extraction solvent. Liquid–liquid extraction is described in more details in the supplement. Lipidomics analysis was performed by the Metabolomics Unit, Technology Centre, Institute for Molecular Medicine Finland (FIMM), University of Helsinki.

### Lipid metabolism data analysis

The lipid composition (%) and concentration from cell pellets (nmol per million cells) and serums (nmol per liter) extracted from the patient and control blood samples were analyzed. Initially, lipids that were detected in less than 70% of the samples were filtered. Subsequently, in the remaining, the missing values resulted by low intensity of the measured lipid was replaced with a low value, that we measured with the *minimum intensity divided by* 2. The values were log_2_ scaled and for visualization they were Pareto scaled.^23^ The R/Bioconductor library limma was used to compare the lipid intensities in the patient cells to those in the controls.^24^ By defining as covariates within the design formula, the analysis was adjusted for the possible biases introduced by the varied sex and age group of the studied individuals. It is worth mentioning that since concentration and composition data for both lipid species and fatty acids (FAs) were available, we ran a comparison analysis at both the concentration and composition levels. However, we continued the analysis with the results from concentration comparison as it detected a greater number of significantly differential lipids. The lipids and FAs with significantly differential concentration were combined and analyzed using Lipid Ontology (ie, LION) enrichment analysis software to detect the enriched lipid classes within the upregulated and downregulated results.^25^ The Benjamini–Hochberg method was used to adjust for multiple testing throughout our analysis.^21^ Finally, by considering an FDR <0.05 cutoff, the significantly differential lipid species and FAs (in the patient samples compared to the controls), together with their enriched lipid ontology annotations, were discovered. This was achieved while preventing any effects that may have been introduced by poor quality data, varied sex and age of the studied individuals, or multiple testing. For the heatmap visualization, the lipid intensities were log2 scaled, adjusted for the age and sex effects using ComBat method (ie, supported by the SVA R/Bioconductor package), and scaled by row (ie, the lipids).^26^

### Statistical analysis

Data are displayed as mean ± SD. A 2-tailed unpaired *t*-test was used to determine significance, with a cut-off point of *p* < .05. To evaluate concordance in gene expression intensities between RNA-seq and qRT-PCR, we first calculated the fold change of each gene between affected patients and healthy controls in qRT-PCR. The calculation of the fold change of each gene in RNA-seq data between patients and controls is described in the “RNA-seq data analysis” section. Linear regression between RNA-seq and RT-qPCR fold-change results was performed. GraphPad Prism version 8.4.2 for Windows (GraphPad Software) was used for statistical analyses.

## Results

### Clinical characteristics

Altogether 6 affected patients with *SGMS2*-related bone disease and 7 healthy controls were included in the study (Table 1). Four patients were heterozygous for p.Arg50*, while 2 patients were heterozygous for either p.Met64Arg or p.Ile62Ser. The patients’ clinical characteristics have been reported previously.^2^^,^^4^ The patients with the p.Arg50* variant included 3 20- to 30-yr-old subjects and 1 older subject. Affected patient 1 had a history of multiple low-energy peripheral fractures and low lumbar spine BMD.^4^ Patient 2 presented with severe childhood-onset primary osteoporosis, low-energy peripheral fractures, vertebral fractures, and a sclerotic cranial lesion, but no nerve palsies or ophthalmological concerns. Patient 3 had spinal and peripheral fractures, low BMD, sclerotic calvarial lesions, recurrent idiopathic peripheral facial nerve palsies (Bell’s palsy), and congenital bilateral glaucoma. Affected patient 4 had a similar skeletal phenotype as well as transient facial, trochlear, and oculomotor nerve palsies with slow recovery.^1^^,^^2^ The patients with *SGMS2* missense variants had a more severe phenotype. Patient 6 with the p.Ile62Ser variant presented bilateral femoral fractures and recurrent transient facial nerve palsies. Patient 5 with the p.Met64Arg variant had spondylometaphyseal dysplasia with marked short stature, severe scoliosis, cranial sclerosis, sensorineural hearing loss, myopia, dilated aortic root, and hypotonia with abnormal myopathic electromyography and facial diplegia.^2^

**Table 1:** Characteristics of the study participants.

**Status**	**Pathogenic variant**	**Age**	**Gender**	**Clinical presentation**	**RNA-seq**	**Lipidomics**	
					**Fibroblasts**	**Fibroblasts**	**Serum**
**Affected 1**	p.Arg50^*^	20-30 yr	Female	Early-onset osteoporosis^4^	Yes	–	–
**Affected 2**	p.Arg50^*^	20-30 yr	Male	Early-onset osteoporosis^2^	Yes	Yes	Yes
**Affected 3**	p.Arg50^*^	50-60 yr	Male	Early-onset osteoporosis^2^	Yes	Yes	Yes
**Affected 4**	p.Arg50^*^	20-30 yr	Female	Early-onset osteoporosis^2^	Yes	Yes	Yes
**Affected 5**	p.Met64Arg	10-20 yr	Male	Infancy-onset skeletal dysplasia^2^	Yes	Yes	-
**Affected 6**	p.Ile62Ser	40-40 yr	Female	Infancy-onset skeletal dysplasia^2^	Yes	Yes	-
**Healthy 1**	–	50-60 yr	Female	Unaffected	Yes	Yes	Yes
**Healthy 2**	–	20-30 yr	Male	Unaffected	Yes	Yes	Yes
**Healthy 3**	–	30-40 yr	Female	Unaffected	Yes	Yes	Yes
**Healthy 4**	–	40-50 yr	Male	Unaffected	Yes	Yes	Yes
**Healthy 5**	–	20-30 yr	Female	Unaffected	Yes	Yes	-
**Healthy 6**	–	20-30 yr	Male	Unaffected	Yes	Yes	-
**Healthy 7**	–	50-60 yr	Male	Unaffected	Yes	–	–

### Significantly differentially-expressed genes

**Figure 1:**
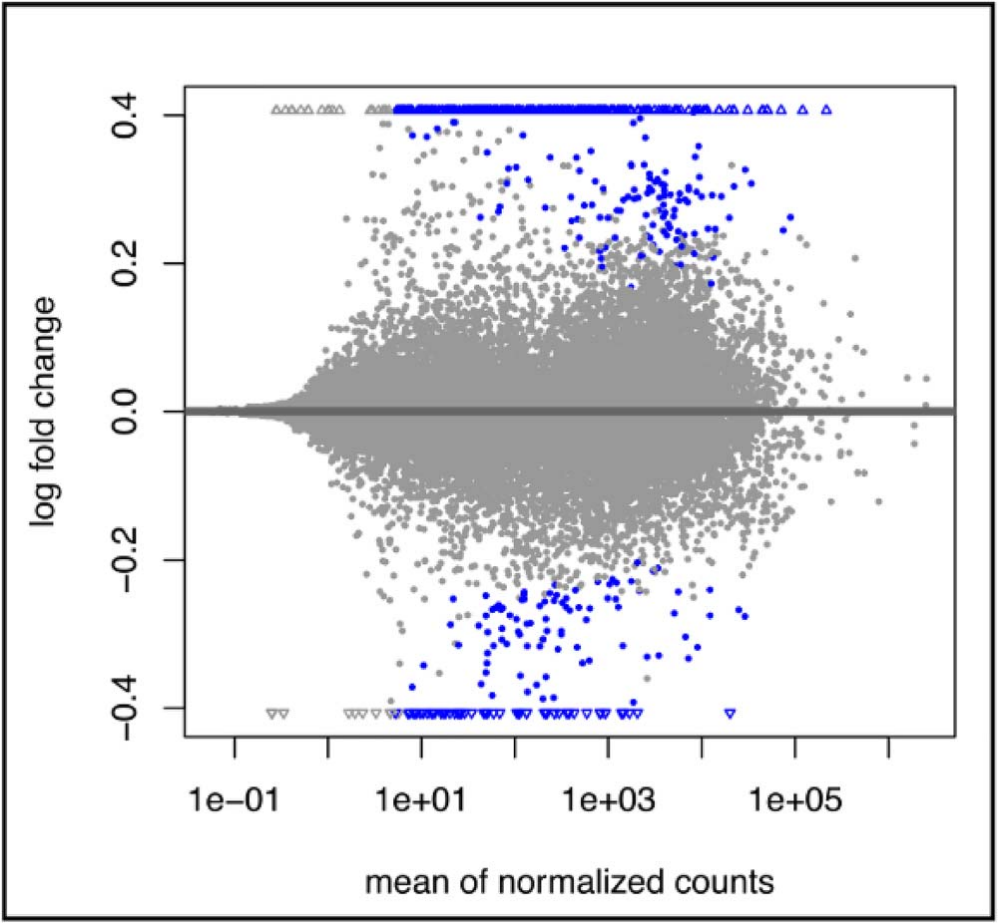
Differential gene expression analysis. (A) Scatterplot visualizing the distribution of the shrunken expression log foldchange of the genes (comparing six patient samples to seven control samples) against the average expression (ie, LCPM) of the genes across all studied samples. The significantly differential genes (FDR < 0.05) are highlighted. A total of 215 significantly up- and 58 significantly down-regulated genes detected in differentially expression analysis of the RNA-seq data.

The differential expression analysis of our RNA-seq data detected 215 significantly up- and 58 significantly downregulated genes in 6 patient samples compared to 7 controls (Figure 1A, Tables S1 and S2). Table 2 summarizes the top 10 most significantly differentially-expressed genes (sorted by FDR in increasing order), up- and downregulated, comparing patients to the controls. The most significantly upregulated gene, cadherin 6 (*CDH6*), encodes a vital cell–cell adhesion protein crucial for nervous system development and maintenance.^27^ Adhesion G Protein-Coupled Receptor G7 (*ADGRG7*), the most significantly downregulated gene, encodes a membrane-bound protein. *ADGRG7* has been implicated as a genetic factor involved in the development of adolescent idiopathic scoliosis, which associates with lower peak bone mass and osteopenia at puberty.^28^

**Table 2:** Top 10 most significantly differentially expressed up- and down-regulated genes in the patient samples compared to the controls.

**Up-regulated**				
**ENSEMBLE**	**Gene**	**Biotype**	**Log2**	**FDR**
**ENSG00000113361.13**	*CDH6*	PC	3.55	7.41E−07
**ENSG00000148677.7**	*ANKRD1*	PC	5.40	1.62E−06
**ENSG00000159251.8**	*ACTC1*	PC	3.62	1.11E−05
**ENSG00000174469.23**	*CNTNAP2*	PC	4.96	1.80E−05
**ENSG00000196711.9**	*ALKAL1*	PC	17.06	3.92E−05
**ENSG00000168427.9**	*KLHL30*	PC	2.88	0.00034
**ENSG00000163017.14**	*ACTG2*	PC	6.89	0.00045
**ENSG00000131737.7**	*KRT34*	PC	3.06	0.00104
**ENSG00000165757.9**	*JCAD*	PC	1.56	0.00114
**ENSG00000204362.6**	*LINC02783*	IncRNA	2.39	0.00114
**Down-regulated**				
**ENSG00000286091.1**	*-*	TEC	−25.24	1.61E−12
**ENSG00000144820.8**	*ADGRG7*	PC	−24.31	2.33E−12
**ENSG00000170627.11**	*GTSF1*	PC	−21.42	3.64E−09
**ENSG00000225968.8**	*ELFN1*	PC	−0.60	0.0026
**ENSG00000079931.15**	*MOXD1*	PC	−1.05	0.0078
**ENSG00000107611.16**	*CUBN*	PC	−0.85	0.0098
**ENSG00000280241.4**	*-*	IncRNA	−1.27	0.0098
**ENSG00000169302.16**	*STK32A*	PC	−0.62	0.0100
**ENSG00000197841.15**	*ZNF181*	PC	−0.34	0.0133
**ENSG00000228801.8**	*PCMTD1-DT*	IncRNA	−0.39	0.0136

FDR < 0.05. Abbreviations: PC, protein coding; IncRNA, long noncoding RNA; TEC, to be experimentally confirmed.

### Significantly enriched biological processes

The significantly differentially-expressed genes in our RNA-seq data were enriched with 169 GO Biological Processes (Table S3). These include angiogenesis, cardiac muscle tissue morphogenesis, several signaling pathways (eg, phosphatidylinositol 3-kinase, ERBB3, platelet-derived growth factor receptor), vascular endothelial cell proliferation, neural precursor cell proliferation, cardiac muscle cell proliferation, cytoskeleton organization, and skeletal myofibril assembly (Table S3). For improved readability, we clustered these enriched GOs based on their semantic similarities. The GO clusters, the similarities of the GO descriptions, and the most common terms in the GO descriptions within each cluster are illustrated in a heatmap included in the supplement (Figure S1).

Based on their clinical relevance, we manually further categorized 81 biological processes (out of the overall 169) into 5 groups (Tables 3 and 4). Out of these, groups 1-4 were created based on processes linked to clinical manifestations diagnosed in patients with *SGMS2*-related skeletal disorder: group 1—skeletal processes, group 2—neurological processes, group 3—ocular processes, and group 4—muscular processes. Furthermore, group 5—membrane processes, alludes to the fact that SMS2 catalyzes the synthesis of SM, which is a crucial compound in the cell membrane.

**Table 3:** GOs included in Groups 1, 2, and 3.

**Group 1: Skeletal processes**	
**GO:0072132**	Mesenchyme morphogenesis
**GO:0014068**	Positive regulation of phosphatidylinositol 3-kinase signaling
**GO:0045736**	Negative regulation of cyclin-dependent protein serine/threonine kinase activity
**GO:0043410**	Positive regulation of MAPK cascade
**GO:0070374**	Positive regulation of ERK1 and ERK2 cascade
**GO:0043406**	Positive regulation of MAP kinase activity
**GO:0032967**	Positive regulation of collagen biosynthetic process
**GO:0001502**	Cartilage condensation
**GO:0032924**	Activin receptor signaling pathway
**GO:0046849**	Bone remodeling
**GO:0010862**	Positive regulation of pathway-restricted SMAD protein phosphorylation
**GO:0007623**	Circadian rhythm
**GO:0010692**	Regulation of alkaline phosphatase activity
**GO:0019221**	Cytokine-mediated signaling pathway
**GO:0045639**	Positive regulation of myeloid cell differentiation
**Group 2: Neuronal processes**	
**GO:0045161**	Neuronal ion channel clustering
**GO:0038129**	ERBB3 signaling pathway
**GO:0097154**	GABAergic neuron differentiation
**GO:0021781**	Glial cell fate commitment
**GO:0038128**	ERBB2 signaling pathway
**GO:0021782**	Glial cell development
**GO:0015820**	Leucine transport
**GO:2000178**	Negative regulation of neural precursor cell proliferation
**GO:0048935**	Peripheral nervous system neuron development
**GO:0048715**	Negative regulation of oligodendrocyte differentiation
**GO:0015844**	Monoamine transport
**GO:1903826**	L-arginine transmembrane transport
**GO:0050965**	Detection of temperature stimulus involved in sensory perception of pain
**GO:0045747**	Positive regulation of Notch signaling pathway
**GO:0007638**	Mechanosensory behavior
**GO:0042423**	Catecholamine biosynthetic process
**GO:1902430**	Negative regulation of amyloid-beta formation
**GO:0048709**	Oligodendrocyte differentiation
**GO:0021756**	Striatum development
**GO:0001964**	Startle response
**GO:0007612**	Learning
**GO:0097150**	Neuronal stem cell population maintenance
**GO:0014015**	Positive regulation of gliogenesis
**GO:0021544**	Subpallium development
**Group 3: Ocular processes**	
**GO:0046533**	Negative regulation of photoreceptor cell differentiation
**GO:0043010**	Camera-type eye development
**GO:0030213**	Hyaluronan biosynthetic process

**Table 4:** GOs included in Groups 4 and 5.

**Group 4: Muscular processes**	
**GO:0045110**	Intermediate filament bundle assembly
**GO:0003215**	Cardiac right ventricle morphogenesis
**GO:0035914**	Skeletal muscle cell differentiation
**GO:1905209**	Positive regulation of cardiocyte differentiation
**GO:0031033**	Myosin filament organization
**GO:0003278**	Apoptotic process involved in heart morphogenesis
**GO:0055006**	Cardiac cell development
**GO:0061302**	Smooth muscle cell-matrix adhesion
**GO:0014866**	Skeletal myofibril assembly
**GO:0006940**	Regulation of smooth muscle contraction
**GO:0031032**	Actomyosin structure organization
**GO:0045823**	Positive regulation of heart contraction
**GO:0003197**	Endocardial cushion development
**GO:0055008**	Cardiac muscle tissue morphogenesis
**GO:0060038**	Cardiac muscle cell proliferation
**GO:2000251**	Positive regulation of actin cytoskeleton reorganization
**GO:0014897**	Striated muscle hypertrophy
**GO:0055010**	Ventricular cardiac muscle tissue morphogenesis
**GO:0003184**	Pulmonary valve morphogenesis
**GO:0061049**	Cell growth involved in cardiac muscle cell development
**GO:0048662**	Negative regulation of smooth muscle cell proliferation
**GO:0010002**	Cardioblast differentiation
**GO:0043500**	Muscle adaptation
**GO:1904706**	Negative regulation of vascular associated smooth muscle cell proliferation
**GO:0042149**	Cellular response to glucose starvation
**GO:0045987**	Positive regulation of smooth muscle contraction
**Group 5: Membrane processes**	
**GO:0007155**	Cell adhesion
**GO:0033630**	Positive regulation of cell adhesion mediated by integrin
**GO:0016339**	Calcium-dependent cell-cell adhesion via plasma membrane cell adhesion molecules
**GO:0001953**	Negative regulation of cell-matrix adhesion
**GO:0007156**	Homophilic cell adhesion via plasma membrane adhesion molecules
**GO:0007043**	Cell–cell junction assembly
**GO:0038063**	Collagen-activated tyrosine kinase receptor signaling pathway
**GO:0098609**	Cell–cell adhesion
**GO:0070831**	Basement membrane assembly
**GO:0034332**	Adherens junction organization
**GO:0060353**	Regulation of cell adhesion molecule production
**GO:1903596**	Regulation of gap junction assembly
**GO:0045216**	Cell–cell junction organization

**Figure 2:**
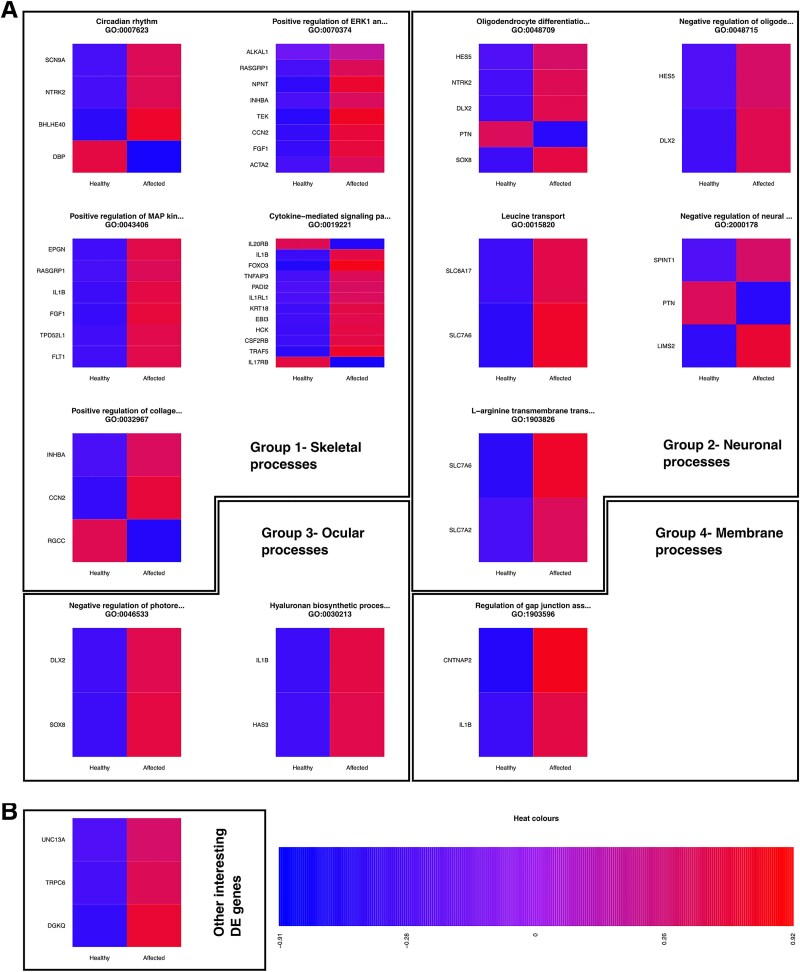
The expression of the significantly differentially expressed genes associated with the categorized significant GO groups. (A) Heatmap illustration of the average expression of the significantly differentially expressed genes (FDR < 0.05) of the RNA-seq data associated with a selection of categorized significant GO groups. The average expressions are scaled by row and shown for the affected patients and healthy controls. (B) Presentation of other interesting significantly differentially expressed genes (FDR < 0.05), comparing patients to controls, that are not included in the five categorized GO-groups. The average log foldchange expressions are scaled by row and shown for the affected patients and healthy controls.

To get an in-depth view of the significantly differentially-expressed genes included in each GO group, the significantly differentially-expressed genes associated with several GO groups are presented in Figure 2A and Figure S3. The heatmaps present the average expression of the significant genes (associated with the GO groups) across the patient and control samples (Figure 2A). It is worth noting that the expression values are adjusted for sex and age and scaled by row (ie, the genes). We also illustrate the expression levels of 3 significantly differentially-expressed genes. Although these genes were not associated with any of the significant GOs in the 4 groups, they were considered relevant based on previous clinical findings in CDL studies (Figure 2B). The groups are presented and discussed in more detail in the discussion, with a special emphasis on the well-known genes and GOs. All significantly differentially-expressed genes associated with the 169 significant GOs discovered are presented in Table S4. The list of all significantly differentially-expressed genes (when comparing patient samples to the control samples) are presented in Figure S2. The vast majority of the differentially-expressed genes that are associated with the significant GOs in the 4 groups (Figure 2A) as well as the other interesting genes (Figure 2B) are upregulated in the patient samples compared to the control samples.

### Significantly enriched pathways

We next performed an overrepresentation analysis to determinate if certain Reactome pathways are enriched in our RNA-seq data. The significantly differentially-expressed (up- and downregulated) genes, with a HUGO Gene Nomenclature Committee (HGNC) symbol were used as the input. In total 140 out of the 254 input genes were found in Reactome, where 705 pathways were hit by at least one of these genes (Table S5). The top 25 pathways are presented in Table 5. Among these pathways, there are several that we will discuss in further detail below, namely, *BMAL1*:*CLOCK*, *NPAS2* activates circadian gene expression (R-HSA-1368108), circadian clock (R-HSA-400253), interleukin-33 signaling (R-HSA-9014843), interleukin-1 processing (R-HSA-448706), interleukin-10 signaling (R-HSA-6783783), inhibition of signaling by overexpressed estimated glomerular filtration rate (EGFR) (R-HSA-5638303), EGFR interacts with phospholipase C-gamma (R-HSA-212718), and electric transmission across gap junctions (R-HSA-112303). Furthermore, 4 enriched pathways that are associated with lipid metabolism were detected, that is, regulation of lipid metabolism by PPARalpha (R-HSA-400206), glycerophospholipid (GPL) biosynthesis (R-HSA-1483206), phospholipid metabolism (R-HSA-1483257), and metabolism of lipids (R-HSA-556833) (Table 5, Table S5).

**Table 5:** Enriched pathways in the RNA-seq dataset. The results of overrepresentation analysis performed on the significantly differentially expressed (up- and downregulated) genes in affected patients compared to healthy controls (FDR < 0.05). Out of the 254 differentially expressed genes (only genes with a HGNC symbol were included), 140 genes were found in Reactome. Furthermore, 705 pathways were associated to at least one of these genes.

**Top 25 most significant pathways**								
**Pathway name**		**Entities**			**Reactions**			
		**Found**	**Ratio**	***p*-value**	FDR*	**Found**	**Ratio**	
**1. BMAL1:CLOCK,NPAS2 activates circadian gene expression**		6/43	0.003	1.85e−04	0.133	6/20	0.001	
2. CLEC7A/inflammasome pathway		3/8	5.17e−04	4.98e−04	0.178	2/4	2.72e−04	
3. Formation of the cornified envelope		9/138	0.009	.001	0.282	9/27	0.002	
4. NOTCH4 intracellular domain regulates transcription		4/26	0.002	.002	0.282	2/9	6.11e−04	
**5. Interleukin-33 signaling**		2/4	2.59e−04	.003	0.318	2/2	1.36e−04	
6. Antagonism of activin by follistatin		2/4	2.59e−04	.003	0.318	2/2	1.36e−04	
**7. Electric transmission across gap junctions**		2/6	3.88e−04	.006	0.489	3/4	2.72e−04	
8. Transmission across electrical synapses		2/6	3.88e−04	.006	0.489	3/4	2.72e−04	
9. Regulation of NPAS4 gene expression		3/22	0.001	.009	0.489	10/11	7.47e−04	
10. Collagen chain trimerization		4/44	0.003	.01	0.489	4/28	0.002	
**11. Inhibition of signaling by overexpressed EGFR**		2/8	5.17e−04	.01	0.489	1/2	1.36e−04	
12. Signaling by overexpressed WT EGFR in cancer		2/8	5.17e−04	.01	0.489	1/2	1.36e−04	
13. Keratinization		10/226	0.015	.011	0.489	16/34	0.002	
14. RUNX3 regulates YAP1-mediated transcription		2/9	5.82e−04	.013	0.489	2/3	2.04e−04	
15. Kidney development		5/75	0.005	.014	0.489	5/50	0.003	
**16. Circadian clock**		6/105	0.007	.015	0.489	6/59	0.004	
17. TP53 regulates transcription of additional cell cycle genes whose exact role in the p53 pathway remain uncertain		3/28	0.002	.016	0.489	3/14	9.51e−04	
18. ECM proteoglycans		5/79	0.005	.017	0.489	6/23	0.002	
19. Estrogen-dependent nuclear events downstream of ESR-membrane signaling		3/29	0.002	.018	0.489	3/12	8.15e−04	
**20. EGFR interacts with phospholipase C-gamma**		2/11	7.11e−04	.019	0.489	3/3	2.04e−04	
21. Regulation of NPAS4 mRNA translation		2/11	7.11e−04	.019	0.489	2/2	1.36e−04	
22. Adrenoceptors		2/11	7.11e−04	.019	0.489	5/12	8.15e−04	
23. Regulation of NPAS4 gene transcription		2/12	7.76e−04	.022	0.489	8/9	6.11e−04	
**24. Interleukin-1 processing**		2/12	7.76e−04	.022	0.489	2/5	3.40e−04	
**25. Interleukin-10 signaling**		5/86	0.006	.024	0.489	1/15	0.001	
**Enriched pathways associated with lipid metabolism**							
**Pathway name**		**Entities**			**Reactions**		
		**Found**	**Ratio**	***p*-value**	FDR*	**Found**	**Ratio**
**561. Regulation of lipid metabolism by PPARalpha**		2 / 177	0.011	.845	0.845	1/45	0.003
**660. Glycerophospholipid biosynthesis**		1/221	0.014	.985	0.985	1/133	0.009
**677. Phospholipid metabolism**		1/314	0.020	.997	0.997	1/218	0.015
**704. Metabolism of lipids**		6/1508	0.098	1	1	12/981	0.067

The most interesting pathways for our study are bolded. FDR*, false discovery rate. *Entities found*: the number of curated molecules that are common between the submitted dataset and the pathway named in column 1. *Entities ratio*: The proportion of Reactome pathway molecules represented by this pathway. *Entities p-value*: The result of the statistical test for over-representation. *Entities FDR*: Corrected over-representation probability. *Reactions found*: The number of reactions in the pathway that are represented by at least one molecule in the submitted dataset. *Reactions ratio*: The proportion of Reactome reactions represented by this pathway.

### RNA splicing pattern between patients and healthy controls in SGMS2 gene

No difference in RNA splicing patterns of the *SGMS2* gene was observed between the patients with heterozygous pathogenic variants c.148C>T (p.Arg50*), c.185T>G (p.Ile62Ser), or c.191T>G (p.Met64Arg) and healthy controls (Figures S4–S6).

### Validity of RNA sequencing

**Figure 3:**
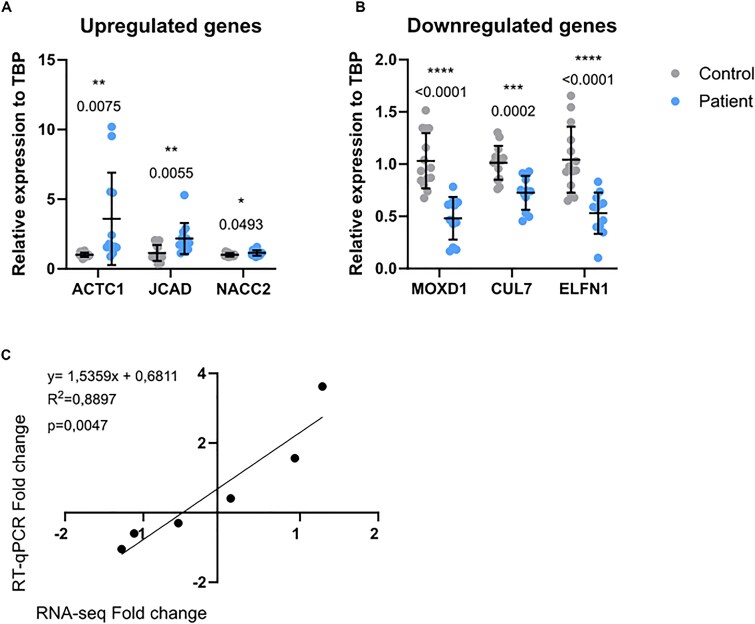
RNA-seq validation implies trustworthy results. **(**A) Validation of transcriptome data by determining by qRT-PCR the relative mRNA expression of upregulated genes: *ACTC1, JCAD, NACC2* and of (B) downregulated genes: *MOXD1*, *CUL7*, and *ELFN1*. The gene expressions were normalized to *TBP* according to the ΔΔCt method. Significant expression was analyzed with unpaired *t*-test, two-tailed (* < 0.05, ** < 0.01, *** < 0.001, **** < 0.0001), mean ± SD (standard deviation) values are presented (C) linear association between gene expression fold-changes measured by RT-qPCR and RNA-seq. Linear regression equation, R2, and *p*-value are displayed.

Messenger RNA expression of 6 target genes, selected from the 25 top up- and down-regulated genes in the dataset, was verified by qRT-PCR. The results confirmed that *ACTC1*, *JCAD*, and *NACC2* were upregulated in fibroblasts of six affected patients compared to the fibroblasts of 7 sex- and age-matched healthy controls, while *MOXD1*, *CUL7*, and *ELFN1* were downregulated (Figure 3A and B). This was further confirmed by the statistically significant association (tested by linear regression) between the RT-qPCR and RNA-seq data (Figure 3C).

### Significantly up- and downregulated lipids

**Figure 4:**
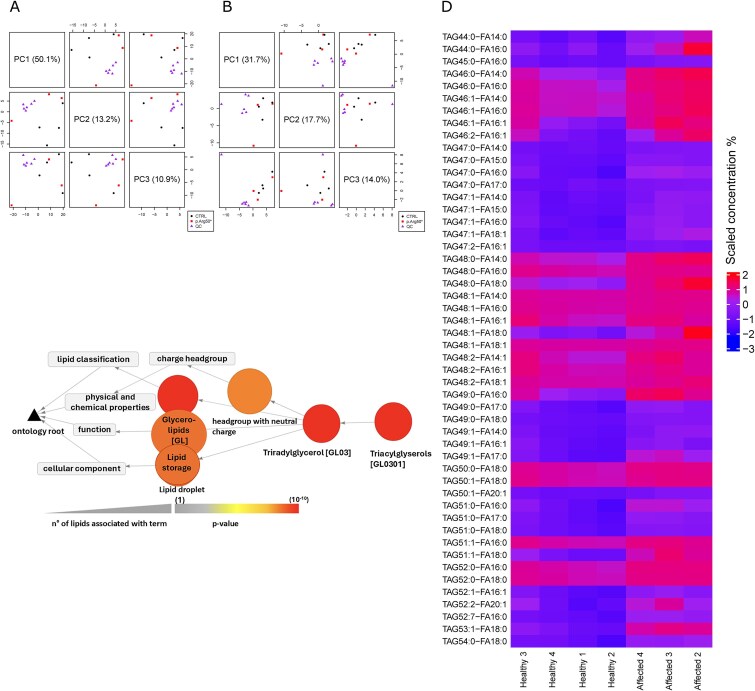
The discovered significant lipid ontology classes. (A and B) Scatterplot matrices illustrate the distribution of the (A) lipid species concentrations and (B) fatty acid concentration in the serum samples based on PC1-PC3. The measurements are Pareto and log2 scaled. The percentage of the data variance explained by the PC dimension is stated in parentheses. (C) The graph illustrates the significantly enriched lipid ontologies within the upregulated lipids (comparing patients to the controls). The interactions between these different lipid ontologies are shown with arrows. The color of the circles within the graph indicates the *p*-values for the enrichment of the ontologies. (D) Heatmap illustrating the intensities of triacylglycerols (TAG) across the serum samples from three affected patients and four healthy controls. The values are log-scaled and row-scaled. They are also adjusted for variances introduced by the sex and age-group differences within the studied individuals. The values of the significantly differential TAGs are shown by applying an FDR <0.05 cutoff.

No significant differences were detected between the lipid levels in the fibroblast pellets of the patients compared to the cell pellets of the controls. Therefore, we hereafter focus on the lipid data of the serum samples, which include samples from 3 individuals with a p.Arg50* mutation and 4 samples from sex- and age-matched controls (Table 1). PCA analysis revealed that the overall levels of lipid species and FA concentration levels of the QC samples did not vary drastically (Figure 4A and B). This supports the reproducibility and reliability of the lipid species and FA measurements. Furthermore, PCA showed that lipid species and FA concentrations in the four control serum samples were more similar to each other, whereas in the patients’ serum samples they were more widely scattered (Figure 4A and B). Overall, 650 lipids (lipid species and FAs detected in 70% of the samples) were identified in the lipidomic analysis and used for differential analysis. The analysis of the lipid values revealed that 55 lipids were upregulated in the serum of the patients (compared to the serum of control individuals), and no lipids were downregulated. The vast majority (50 out of 55) of the upregulated lipids were triacylglycerols (ie, TAGs) (Figure 4D, Table S6). Furthermore, these upregulated lipids mostly comprised of completely saturated (with 0 double bonds) (*n* = 22) or monounsaturated (with one double bond) (*n* = 21) species, while only a few species (*n* = 7) with two or more double bonds was upregulated. Lipid Ontology (ie, LION) enrichment analysis showed biological features, such as lipid droplets (LDs) (LION:0012084) and lipid storage (LION:0012011), were enhanced in patient serum samples compared to controls (Figure 4C, Table S7). We further investigated if the genes associated with TAGs regulation, such as acetyl-Coa carboxylases, acyl-CoA monoacylglycerol acyltransferases, FA desaturases, FA elongases, FA synthase, fatty acyl activating long-chain acyl-CoA synthases, or TAG-hydrolyzing enzymes were differentially expressed in the patient cells compared to the controls (Table S8). However, the expression of none of these genes showed significant differences between the groups (FDR < 0.05) (Table S8).

## Discussion

This study aims to enhance our understanding of pathological mechanisms of CDL by exploring the impact of *SGMS2* variants on the transcriptome and the lipidome in fibroblasts and serum samples, respectively. The most essential findings are discussed in detail below, and the function of the specific genes is described in more detail in the supplement.

### SGMS2 gene splicing patterns between the patients and healthy controls

Exonic variants present some of the strongest links between genotypes and phenotypes. The splicing patterns of pre-mRNAs significantly influence phenotypes by modulating the production and quantity of protein variants.^29^ A heterozygous *SGMS2* pathogenic variant, c.148C>T (p.Arg50*) causes childhood-onset osteoporosis with low BMD and skeletal fragility with or without sclerotic doughnut-shaped lesions. Heterozygous *SGMS2* missense variants c.185T>G (p.Ile62Ser) and c.191T>G (p.Met64Arg) result in a more severe phenotype with neonatal fractures, severe short stature, and spondylometaphyseal dysplasia.^2^ This study found no differences in the RNA splicing patterns between patients with *SGMS2* variants and healthy controls. The phenotypic differences observed are thus attributed to loss-of-function or missense mutations rather than RNA splicing patterns.^2^ The p.Arg50* variant produces a premature stop codon in exon 2, leading to a truncated enzyme, whereas the missense variants c.185T>G and c.191T>G block the export of functional enzymes from the ER, thereby increasing de novo SM production.^2^ This explains why missense mutations in *SGMS2* result in a more severe phenotype than loss-of-function mutations.

### Central skeletal processes in CDL

Calvarial doughnut lesions with bone fragility primarily manifest as a skeletal disorder, presenting with, for example, childhood-onset osteoporosis, low BMD, skeletal fragility, and distinctive skull lesions.^5^ One discovered GO in the transcriptomic dataset is circadian rhythm (GO:0007623) with observed downregulation of *DBP* and upregulation of *BHLHE40* in patients compared to controls (Figure 2A, Figure S3). Circadian clock plays a crucial role in the biological processes of bone cells and bone homeostasis.^30^ In line with this finding, Reactome analysis detected *BMAL1:CLOCK*, *NPAS2 activates circadian gene expression* (R-HSA-5660668) and *circadian clock* (R-HSA-400253) as abnormally regulated pathways in the patients (Table 5). In bone metabolism, the circadian clock regulates osteoblastic differentiation through interactions with both BMP and WNT pathways and transcription factors, such as RUNX2 and OSX. Simultaneously, the circadian rhythm regulates the differentiation of osteoclasts by influencing the RANKL/OPG ratio.^30^ Disruptions in the circadian rhythm can therefore have negative effects on bone turnover and bone matrix mineralization, in line with skeletal features seen in patients with *SGMS2*-related osteoporosis.

The significant positive regulation of the GOs ERK1/ERK2 cascade (GO:0070374) and MAP kinase activity (GO:0043406) reveals alterations in the ERK-MAPK pathway, which has been reported to be associated with various skeletal disorders.^31^ Upregulation of genes like *FGF1*, *NPNT*, *FLT1*, and *IL1B* (Figure 2A, Figure S3) indicated a complex interplay between bone formation and resorption. One noteworthy osteoclast-associated significant GO is the cytokine-mediated signaling pathway (GO:0019221), which is also linked to the upregulation of the interleukin genes *IL1RL1* and *IL1B* (Figure 2A, Figure S3). Correspondingly, Reactome analysis detected *interleukin-1 processing* (R-HSA-448706), *interleukin-33 signaling* (R-HSA-9014843), and interleukin-10 signaling (R-HSA-6783783) as abnormally regulated pathways in the patients (Table 5). Interleukin-1 is known to stimulate the production of other cytokines that activate osteoclasts, causing bone tissue breakdown.^32^ However, bone marrow osteoclast precursors respond differently to IL1B in terms of proliferation and bone resorption.^33^ Interleukin-33 signaling, regulated by IL1RL1, and IL-10 signaling have also been shown to inhibit bone resorption.^34^^,^^35^ Although the reduced bone volume observed in patients with CDL may suggest elevated bone resorption, our findings do not support this. A previous study also found no differences between osteoclast morphology or resorptive capacity in patients with CDL compared to controls.^2^ Interestingly, Reactome analysis detected *inhibition of signaling by overexpressed EGFR* (R-HSA-5638303) and *EGFR interacts with phospholipase C-gamma* (R-HSA-212718) as abnormally regulated pathways in patients (Table 5). Estimated glomerular filtration rate negatively regulates mTOR signaling in bone development, impacting osteoblast differentiation, that is, EGFR-deficient mice exhibit bone defects and impaired ossification.^36^ Phospholipase C enzymes also play a crucial role in osteoblasts, related to metabolic activity and calcium signaling.^37^ This would indicate that the osteoblast rather than osteoclast function is modulated in patients with CDL.

Finally, the significantly-enriched GO:0032967 (positive regulation of collagen biosynthetic process) (Figure 2A) is an intriguing candidate process since unorganized collagenous network has been reported in CDL patients.^6^ One hypothesis is that *SGMS2* variants could impair the formation of secretory vesicles containing pro-collagen (produced in ER) due to the rigidifying effect of SM on both leaflets of the ER bilayer caused by misdirected SM synthesis.^5^^,^^8^ This would interfere with bone development by preventing collagen from being exported properly from the ER. The decrease in *RGCC* and increase in *CCN2* and *INHBA* expression (Figure 2A, Figure S3) imply an effort to compensate for collagen production in patients, which could result in an aberrant collagen network in the bone.

### Possible effects of SGMS2 pathogenic variants in neurons, eyes, and muscle tissue

Neurological symptoms are common in *SGMS2*-related skeletal disorders, particularly in severe cases, and include cranial nerve palsies, ataxia, reduced reflexes, and Alzheimer’s disease.^2^^,^^5^ Studies suggest *SGMS2* variants to disrupt SM metabolism at the plasma membrane rather than to reduce SM synthesis.^2^^,^^5^^,^^8^ The myelin in the brain, rich in SM, insulates nerve cell axons, and is produced by oligodendrocytes, a type of glial cell.^38^ Our study suggested altered glial cell development (GO:0021782) and increased gliogenesis (GO:0014015) in patients versus controls (Table 3), indicating glial cell impact in CDL. Normally, oligodendrocytes can remyelinate disrupted myelin sheaths.^39^ However, patients showed significant changes in expression patterns in oligodendrocyte differentiation (GO:0048709) and its negative regulation (GO:0048715) (Figure 2A), with upregulation of *NTRK2*, *SOX8*, *DLX2*, and downregulation of *PTN* (Figure 2A, Figure S3). This suggests that abnormal oligodendrocytes may in CDL hinder remyelination, exacerbating myelin damage caused by *SGMS2* variants. Oligodendrocyte precursor cell (OPC) development depends on neuronal activity.^39^ GO:2000178 (negative regulation of neural precursor cell proliferation) dysregulation indicates reduced neuronal precursor proliferation in patients, with *PTN* downregulated (Figure 2A, Figure S3). Upregulation of *HES5* in patients (Table S1, Figure S3) suggests impaired neuronal development affecting oligodendrocytes, reducing myelin repair and contributing to neurological symptoms. Neurons and OPCs communicate via glutamatergic and GABAergic synapses, influencing OPC proliferation in demyelination.^39^ Patients showed upregulation of *SLC7A2*, *SLC6A17*, and *SLC7A6*, linked to GO:0015820 (leucine transport) and GO:1903826 (L-arginine transmembrane transport) (Figure 2A, Figure S3), indicating possible changes in oligodendrocyte proliferation and differentiation in *SGMS2*-related disorders. These findings suggest that *SGMS2* variants may affect neuronal support cells, causing axonal demyelination and hindering remyelination due to negative regulation of oligodendrocyte differentiation.

Less typical presentations of *SGMS2*-related skeletal disorders include myopia, glaucoma, and muscle function deficits.^2^^,^^3^^,^^5^ Studies indicate that myopic eyes show photoreceptor degeneration, leading to visual impairment,^40^ and hyaluronic acid concentration correlates with various glaucoma types.^41^^,^^42^ Our analysis identified negative regulation of photoreceptor cell differentiation (GO:0046533), upregulation of *SOX8* and *DLX2*, regulation of the hyaluronan biosynthetic process (GO:0030213), and increased expression of *HAS3* and *IL1B* in patients compared to controls (Figure 2A, Figure S3). These results imply inadequate photoreceptor development and potential hyaluronic acid upregulation, contributing to myopia and glaucoma in some CDL patients. Notably, upregulation of *MYOC* in patients was observed (Table S1, Figure S3). Consistently, *MYOC* mutations are linked to juvenile open-angle glaucoma.^43^ Although muscle function deficits are rare in CDL, described thus far in only 2 patients,^3^ our analysis revealed multiple GO terms and differentially expressed genes associated with muscle processes (Table 4, Tables S1–S3). The interconnected nature of the musculoskeletal system suggests that muscle processes may be more impacted in CDL patients than previously recognized.

### Membrane processes and lipid data

Significant GOs related to membrane processes are intriguing, given SMS2’s role in catalyzing SM synthesis. Our analysis revealed several membrane-related GOs (Table 4). Particularly, GO:1903596 (regulation of gap junction assembly) and the upregulation of *IL1B* are of great interest (Figure 2A, Figure S3), since Reactome analysis also detected Electric Transmission Across Gap Junctions (R-HSA-112303) as a regulated pathway (Table 5). Gap junctions, intercellular channels connecting the cytoplasmic compartments of neighboring cells, play vital roles in electrical synapses between neurons.^44^ Intriguingly, gap junctions also play an important role in bone tissue by transducing mechanical signals throughout bone cell networks.^45^ Gap junction formation is regulated partly by mechanical stimulation and gap junctions in osteocytes respond to these stimuli resulting in bone formation or resorption.^46^ Mäkitie et al. have shown in bone biopsies from 2 patients with the p.Arg50* variant that the osteocyte canalicular network is distorted, suggesting that the signaling system within the bone is not fully functional.^6^ This suggests that *SGMS2* variants may disrupt gap junction assembly in the membrane, affecting bone homeostasis as well as the transmission of cell–cell information across various tissues.

Additional upregulated differentially expressed genes observed in patients compared to controls are *DGKQ*, *TRPC6*, and *UNC13A* (Figure 2B, Figure S3). These genes are linked to DAG, the byproduct produced during SM synthesis,^47–49^ suggesting a potential alteration in DAG concentration in patients with CDL. However, our data, as well as our previous study, failed to detect this.^2^ Instead, our lipidomics analysis revealed elevated levels of TAGs in patients with the p.Arg50* variant compared to controls (Table S6). This may suggest a rapid conversion of the accumulated DAG into TAG in S*GMS2*-related skeletal disorders, as previously hypothesized.^2^ However, we discovered no significant differences between patients and controls (Table S8) in the expression levels of genes associated with TAG regulation or in pathways involved in TAG metabolism (Table S5). Instead, Reactome analysis detected four pathways associated with lipid metabolism (Table 5). Glycerophospholipid biosynthesis (R-HSA-1483206) is a promising finding since Sokaya and colleagues have shown in an earlier study that SMS2 missense variants are associated with imbalances in GPL profile due to aberrant SM distributions.^8^ Even though the effect of p.Arg50* variant on the GPL profile was not specified in this study, one could speculate that since p.Arg50* also induces aberrant distribution of SM,^8^ the GPL profile of these cells may be affected. Our gene expression data suggests that the GPL biosynthesis may be altered in patients with the p.Arg50* variant.

Finally, our lipidomic data implies that the observed changes primarily target storage lipids, particularly TAGs (completely saturated and monounsaturated), rather than affecting overall FA synthesis, elongation, or desaturation processes (Table S6). LION enrichment analysis (Figure 4C, Table S7) of our serum lipidomics data also detected lipid storage (LION:0012011) and LD (LION:0012084) as cellular features differentially regulated in patients compared to controls. Changes in LDs’ properties, may indicate alterations in cellular dynamics. Ginsberg et al.^50^ highlights the importance of lipoproteins in lipid transport rather than free LDs in the blood, particularly the role of TAG-rich lipoprotein particles and LDL particles containing cholesterol esters, which are essential for energy storage and cellular signaling. The observed dysregulation of lipid storage and droplet formation may suggest potential disruptions in normal lipoprotein metabolism. Interestingly, we also detected an upregulation of *CYP2E1* and *FOXO3*, suggesting a potential mechanism for altered lipid dynamics in the patients (Table S1, Figure S3). They may play roles in pathways that are involved in lipid dysregulation in patients with CDL.

### Study limitations and conclusion

We acknowledge several study limitations. Due to challenges in obtaining preferred materials, that is, native osteoblasts or mesenchymal stem cells, we used fibroblasts and serum samples from patients with CDL. It would have been beneficial to include cell lines from various tissues like bone, muscle, and eye. Therefore, we acknowledge that it is possible that our model system does not reliably reflect expression profiles in the physiological conditions. Additionally, RNA-seq data was limited to only a few patient samples per *SGMS2* variant. Larger number of study participants as well as technical replicates would have been preferable. In the lipidomics analysis, no differentially abundant lipids were detected in the analysis of the fibroblast samples, while analysis of serum samples showed upregulation of TAGs, albeit based on a small subset of patients. Finally, we acknowledge that no confirmatory experiments were performed to future follow up our findings.

While we cannot directly correlate our findings from fibroblast and serum samples with the CDL pathology in bone or other tissues, our transcriptomics and lipidomics results offer valuable insights into gene expression, lipid composition, and cellular pathways related to CDL. Importantly, the main goal of this study is to lay the groundwork for future investigations and sheds light on potential molecular mechanisms underlying this complex skeletal disorder, guiding future functional studies on CDL.

## Ethics approval statement and patient consent statement

The study was authorized by the Helsinki University Hospital’s Ethical Committee; research permit: HUS/265/2023, ethical permission: HUS/404/2018. All study participants or their guardians have signed an informed consent. Personal information and all omics data have been processed in accordance with EU’s General Data Protection Regulation (GDPR).

## Supplementary Material

Supplement_ziaf128

Supplement_tables_1-8_ziaf128

## Data Availability

The processed data from RNA-sequencing analysis and lipidomics analysis are included in the supplement. The raw data of these analyses are available from the corresponding author upon reasonable request.
