# Supplementary material for: Transcriptomic and lipidomic profiling provide novel insight into the pathogenesis of monogenic SGMS2-related osteoporosis
Source: JBMR Plus. 2025 Aug 13;9(10):ziaf128. doi: 10.1093/jbmrpl/ziaf128 (PMC12445838; doi:10.1093/jbmrpl/ziaf128)
Supplement: Supplement_ziaf128 [file supplement_ziaf128.pdf]

Transcriptomic and lipidomic profiling provide novel insight into the pathogenesis of a rare monogenic form of osteoporosis

Sandra Pihlström[1,2]†, Ali Oghabian[1,2] †, Kirsi Määttä[1,2], Jelmer Legebeke[3] Riikka E. Mäkitie[1,2,4], Philippe M Campeau[5], Pauline A Terhal[6], Lorenzo D Botto[7], Vesa M. Olkkonen[8,9], Outi Mäkitie[1,2,3,10]††, Minna Pekkinen[1,2,10]††*

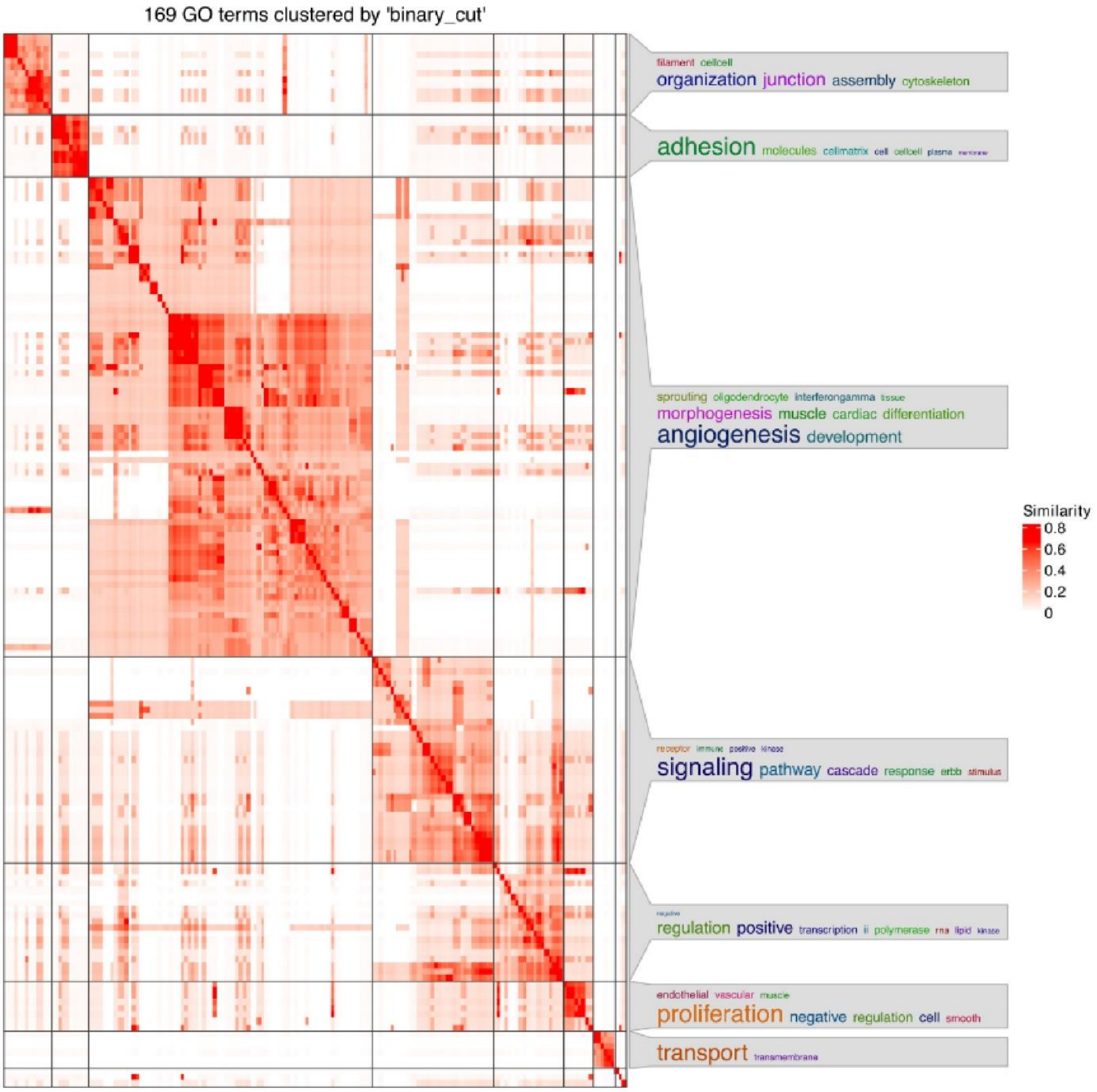

Supplement figure S1. Significantly enriched Gene Ontologies. The Gene Ontology (GO) Biological Process categories enriched in the significantly differentially expressed genes were extracted using topGO. In total, 169 GO terms were significantly enriched. SimplifyEnrichment R/Bioconductor package were used to group the GO categories based on their semantic similarities. The size of each term written in the grey box on the right side of the plot describe how frequently the term is used in the corresponding GOs.

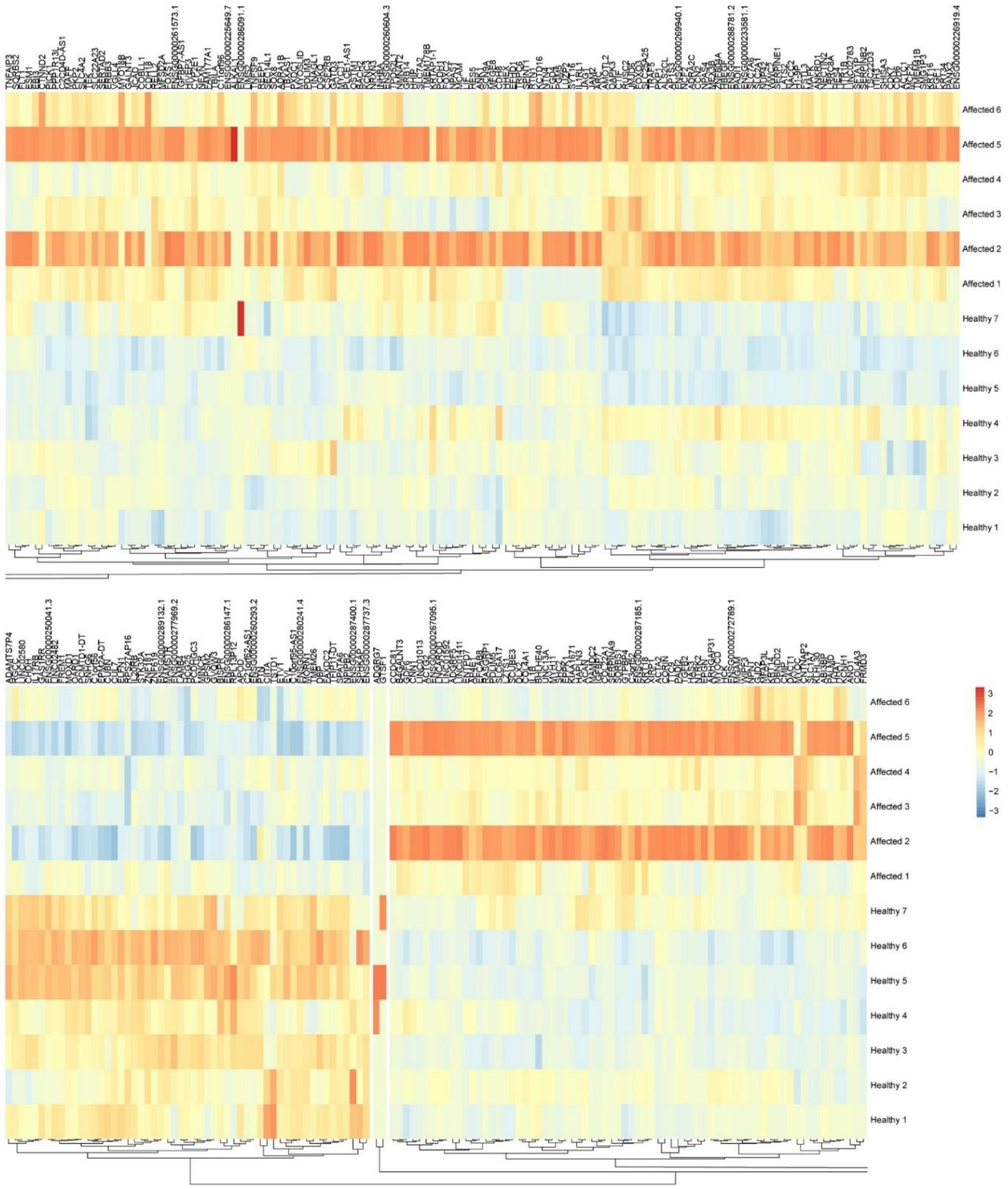

Supplement figure S2. Significantly differentially expressed genes. All significantly differentially expressed genes (FDR<0.05) in the patient samples compared to the control samples are presented in the Spearman heat plot. The expression of a gene is presented separately for each sample: control samples (healthy 1-7) and patient samples (affected 1-6).

**Skeletal processes**		
**Gene**	**Reg.**	**Function**
DBP	↓	Involved in the regulation of circadian rhythm genes (1)
BHLHE40	↑	Negatively regulating the activity of the clock genes and clock-controlled genes (2)
FGF1	↑	Promotes osteogenic differentiation in vitro (3). FGF signaling has shown to inhibits expression of alkaline phosphatase and blocks mineralization (4)
NPNT	↑	Regulates osteoblast differentiation and mineralization (5)
FLT1	↑	Affect bone metabolism, by promoting osteogenesis through PI3K/AKT and MAPK pathways (6)
IL1B	↑	Bone resorption stimulant that increases osteoclastogenesis in vivo and in vitro by upregulating RANKL, resulting in bone resorption (7)<br>Bone marrow osteoclast precursors respond differently to IL1B in terms of proliferation, multinucleation, lifespan, and bone resorption (8)
IL1RL1	↑	Regulates IL-33 signaling which inhibits bone resorption (9)
RGCC	↓	Allows for collagen accumulation (10)
CCN2	↑	Promotes collagen deposition (11)
INHBA	↑	enhances collagen production (12)
**Neuronal processes**		
**Gene**	**Reg.**	**Function**
NTRK2	↑	Inhibits glial differentiation (13)
SOX8	↑	Maintaining the myelinated state (14)
DLX2	↑	Reprograms oligodendrocyte precursor cells into GABAergic inhibitory neurons (15)
PTN	↓	Promotes reparative remyelination (16, 17)<br>Crucial role in neuronal maturation (18)
HES5	↑	Negative regulator of neurogenesis (19)
SLC7A2	↑	Mediates arginine transport (20)
SLC6A17	↑	Mediates arginine transport (21)
SLC7A6	↑	Transports leucine (22)
**Ocular processes**		
**Gene**	**Reg.**	**Function**
SOX8	↑	SOX8 suppression is required for photoreceptor specification in mice (23)
DLX2	↑	Upon loss of Dlx1 and/or Dlx2, some retinal progenitors may commit to photoreceptors in mice (24)
HAS3	↑	Stimulate hyaluronan synthesis (25)
IL1B	↑	Stimulate hyaluronan synthesis (26)
MYOC	↑	Mediates oligodendrocyte differentiation and is involved in optic nerve myelination in mice (27)
**Membrane processes**		
**Gene**	**Reg.**	**Function**
DGKQ	↑	Converts DAG into phosphatidic acid (28)
TRPC6	↑	Proteins that bind DAG (29)
UNC13A	↑	Proteins that bind DAG (30)
IL1B	↑	Negatively impact gap junction assembly (31)
CYP2E1	↑	Promoters of lipid droplet accumulation (32)
FOXO3	↑	Promoters of lipid droplet accumulation (33)
Reg. = regulation in patients compared to controls, ↑/↓ = up- or down-regulated		

Supplement figure S3. Genes associated with bone, neuronal, ocular, and membrane processes. The panel presents genes and their function that are up- or downregulated in fibroblasts from patients with CDL compared with fibroblasts from healthy controls.

References for Supplement figure S3

1. Qin Y, Chen Z-h, Wu J-J, Zhang Z-Y, Yuan Z-D, Guo D-Y, et al. Circadian clock genes as promising therapeutic targets for bone loss. Biomedicine & Pharmacotherapy. 2023;157:114019.
2. Honma S, Kawamoto T, Takagi Y, Fujimoto K, Sato F, Noshiro M, et al. Dec1 and Dec2 are regulators of the mammalian molecular clock. Nature. 2002;419(6909):841-4.
3. Knaup I, Symmank J, Bastian A, Neuss S, Pufe T, Jacobs C, et al. Impact of FGF1 on human periodontal ligament fibroblast growth, osteogenic differentiation and inflammatory reaction in vitro. Journal of Orofacial Orthopedics / Fortschritte der Kieferorthopädie. 2022;83(1):42-55.
4. Mansukhani A, Bellosta P, Sahni M, Basilico C. Signaling by fibroblast growth factors (FGF) and fibroblast growth factor receptor 2 (FGFR2)-activating mutations blocks mineralization and induces apoptosis in osteoblasts. J Cell Biol. 2000;149(6):1297-308.
5. Sun Y, Kuek V, Qiu H, Tickner J, Chen L, Wang H, et al. The emerging role of NPNT in tissue injury repair and bone homeostasis. J Cell Physiol. 2018;233(3):1887-94.
6. Xu K, Fei W, Gao W, Fan C, Li Y, Hong Y, et al. SOD3 regulates FLT1 to affect bone metabolism by promoting osteogenesis and inhibiting adipogenesis through PI3K/AKT and MAPK pathways. Free Radical Biology and Medicine. 2024;212:65-79.
7. Ruscitti P, Cipriani P, Carubbi F, Liakouli V, Zazzeroni F, Di Benedetto P, et al. The role of IL-1β in the bone loss during rheumatic diseases. Mediators Inflamm. 2015;2015:782382.
8. Cao Y, Jansen IDC, Sprangers S, Stap J, Leenen PJM, Everts V, et al. IL-1β differently stimulates proliferation and multinucleation of distinct mouse bone marrow osteoclast precursor subsets. Journal of Leukocyte Biology. 2016;100(3):513-23.
9. Schulze J, Bickert T, Beil FT, Zaiss MM, Albers J, Wintges K, et al. Interleukin-33 is expressed in differentiated osteoblasts and blocks osteoclast formation from bone marrow precursor cells. Journal of Bone and Mineral Research. 2011;26(4):704-17.
10. Luzina IG, Rus V, Lockatell V, Courneya JP, Hampton BS, Fishelevich R, et al. Regulator of Cell Cycle Protein (RGCC/RGC-32) Protects against Pulmonary Fibrosis. Am J Respir Cell Mol Biol. 2022;66(2):146-57.
11. Heng EC, Huang Y, Black SA, Jr., Trackman PC. CCN2, connective tissue growth factor, stimulates collagen deposition by gingival fibroblasts via module 3 and alpha6- and beta1 integrins. J Cell Biochem. 2006;98(2):409-20.
12. Nagaraja AS, Dood RL, Armaiz-Pena G, Kang Y, Wu SY, Allen JK, et al. Adrenergic-mediated increases in INHBA drive CAF phenotype and collagens. JCI Insight. 2021;6(7).
13. Roussel-Gervais A, Sgroi S, Cambet Y, Lemeille S, Seredenina T, Krause KH, et al. Genetic knockout of NTRK2 by CRISPR/Cas9 decreases neurogenesis and favors glial progenitors during differentiation of neural progenitor stem cells. Front Cell Neurosci. 2023;17:1289966.
14. Turnescu T, Arter J, Reiprich S, Tamm ER, Waisman A, Wegner M. Sox8 and Sox10 jointly maintain myelin gene expression in oligodendrocytes. Glia. 2018;66(2):279-94.
15. Boshans LL, Soh H, Wood WM, Nolan TM, Mandoiu II, Yanagawa Y, et al. Direct reprogramming of oligodendrocyte precursor cells into GABAergic inhibitory neurons by a single homeodomain transcription factor Dlx2. Scientific Reports. 2021;11(1):3552.
16. Kuboyama K, Fujikawa A, Suzuki R, Noda M. Inactivation of Protein Tyrosine Phosphatase Receptor Type Z by Pleiotrophin Promotes Remyelination through Activation of Differentiation of Oligodendrocyte Precursor Cells. J Neurosci. 2015;35(35):12162-71.
17. Kuboyama K, Fujikawa A, Suzuki R, Tanga N, Noda M. Role of Chondroitin Sulfate (CS) Modification in the Regulation of Protein-tyrosine Phosphatase Receptor Type Z (PTPRZ) Activity:

PLEIOTROPHIN-PTPRZ-A SIGNALING IS INVOLVED IN OLIGODENDROCYTE DIFFERENTIATION. J Biol Chem. 2016;291(35):18117-28.
18. Wang X. Pleiotrophin: Activity and mechanism. Adv Clin Chem. 2020;98:51-89.
19. Fior R, Henrique D. A novel hes5/hes6 circuitry of negative regulation controls Notch activity during neurogenesis. Dev Biol. 2005;281(2):318-33.
20. Yeramian A, Martin L, Serrat N, Arpa L, Soler C, Bertran J, et al. Arginine transport via cationic amino acid transporter 2 plays a critical regulatory role in classical or alternative activation of macrophages. J Immunol. 2006;176(10):5918-24.
21. Parra LA, Baust T, El Mestikawy S, Quiroz M, Hoffman B, Haflett JM, et al. The orphan transporter Rxt1/NTT4 (SLC6A17) functions as a synaptic vesicle amino acid transporter selective for proline, glycine, leucine, and alanine. Mol Pharmacol. 2008;74(6):1521-32.
22. Rotoli BM, Barilli A, Visigalli R, Ferrari F, Dall'Asta V. y+LAT1 and y+LAT2 contribution to arginine uptake in different human cell models: Implications in the pathophysiology of Lysinuric Protein Intolerance. J Cell Mol Med. 2020;24(1):921-9.
23. Muto A, Iida A, Satoh S, Watanabe S. The group E Sox genes Sox8 and Sox9 are regulated by Notch signaling and are required for Müller glial cell development in mouse retina. Experimental Eye Research. 2009;89(4):549-58.
24. de Melo J, Du G, Fonseca M, Gillespie L-A, Turk WJ, Rubenstein JLR, et al. Dlx1 and Dlx2 function is necessary for terminal differentiation and survival of late-born retinal ganglion cells in the developing mouse retina. Development. 2005;132(2):311-22.
25. Sayo T, Sugiyama Y, Takahashi Y, Ozawa N, Sakai S, Ishikawa O, et al. Hyaluronan synthase 3 regulates hyaluronan synthesis in cultured human keratinocytes. J Invest Dermatol. 2002;118(1):43-8.
26. Wong YK, Tang KT, Wu JC, Hwang JJ, Wang HS. Stimulation of hyaluronan synthesis by interleukin-1beta involves activation of protein kinase C betaII in fibroblasts from patients with Graves' ophthalmopathy. J Cell Biochem. 2001;82(1):58-67.
27. Kwon HS, Nakaya N, Abu-Asab M, Kim HS, Tomarev SI. Myocilin is involved in NgR1/Lingo-1-mediated oligodendrocyte differentiation and myelination of the optic nerve. J Neurosci. 2014;34(16):5539-51.
28. Cai J, Abramovici H, Gee SH, Topham MK. Diacylglycerol kinases as sources of phosphatidic acid. Biochim Biophys Acta. 2009;1791(9):942-8.
29. Dietrich A, Kalwa H, Rost BR, Gudermann T. The diacylgylcerol-sensitive TRPC3/6/7 subfamily of cation channels: functional characterization and physiological relevance. Pflügers Archiv. 2005;451(1):72-80.
30. Topham MK. Signaling roles of diacylglycerol kinases. J Cell Biochem. 2006;97(3):474-84.
31. John GR, Scemes E, Suadicani SO, Liu JS, Charles PC, Lee SC, et al. IL-1beta differentially regulates calcium wave propagation between primary human fetal astrocytes via pathways involving P2 receptors and gap junction channels. Proc Natl Acad Sci U S A. 1999;96(20):11613-8.
32. Wu D, Wang X, Zhou R, Cederbaum A. CYP2E1 enhances ethanol-induced lipid accumulation but impairs autophagy in HepG2 E47 cells. Biochem Biophys Res Commun. 2010;402(1):116-22.
33. Wang L, Zhu X, Sun X, Yang X, Chang X, Xia M, et al. FoxO3 regulates hepatic triglyceride metabolism via modulation of the expression of sterol regulatory-element binding protein 1c. Lipids in Health and Disease. 2019;18(1):197.

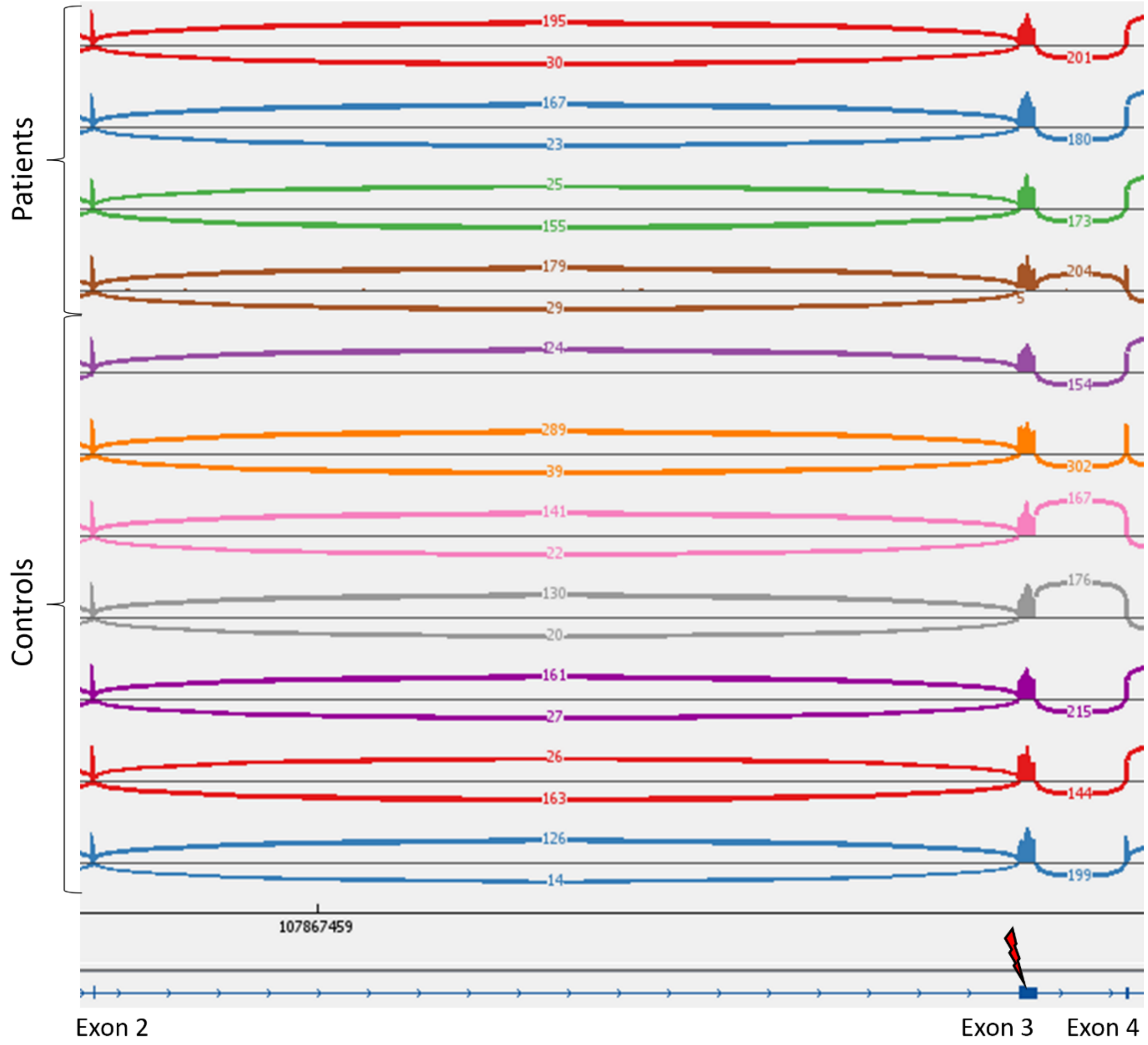

Supplement figure S4. Alternative splicing analysis between the four patients with the p.Arg50* variant and controls. No splicing differences were seen between the patient and control samples. The red lightning bolt indicates the exon with the p.Arg50* variant. The reference transcript used was NM_001375905.

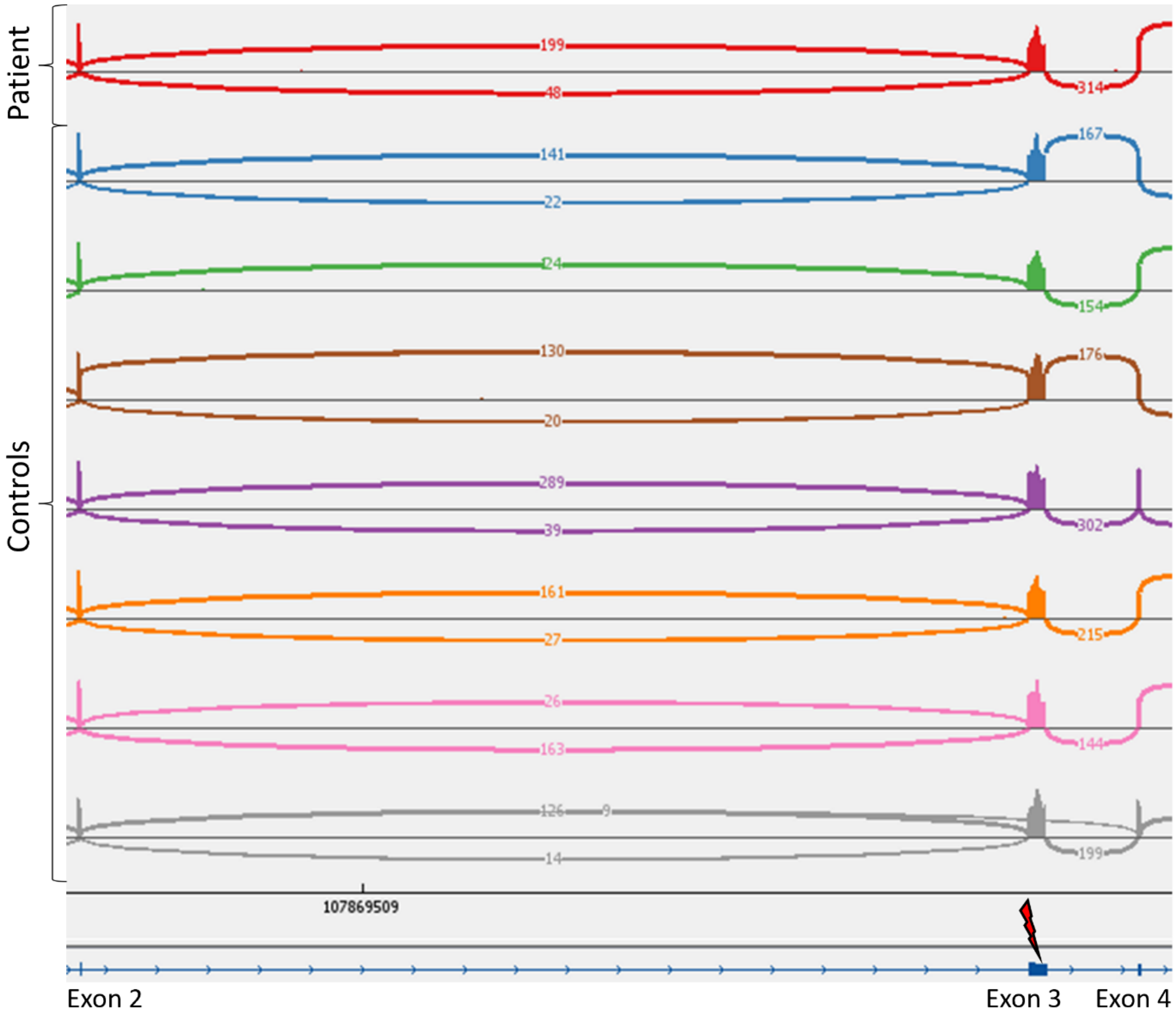

Supplement figure S5. Alternative splicing analysis between the patient with the p.Ile62Ser variant and controls. No splicing differences were seen between the patient and control samples. The red lightning bolt indicates the exon with the p.Ile62Ser variant. The reference transcript used was NM_001375905.

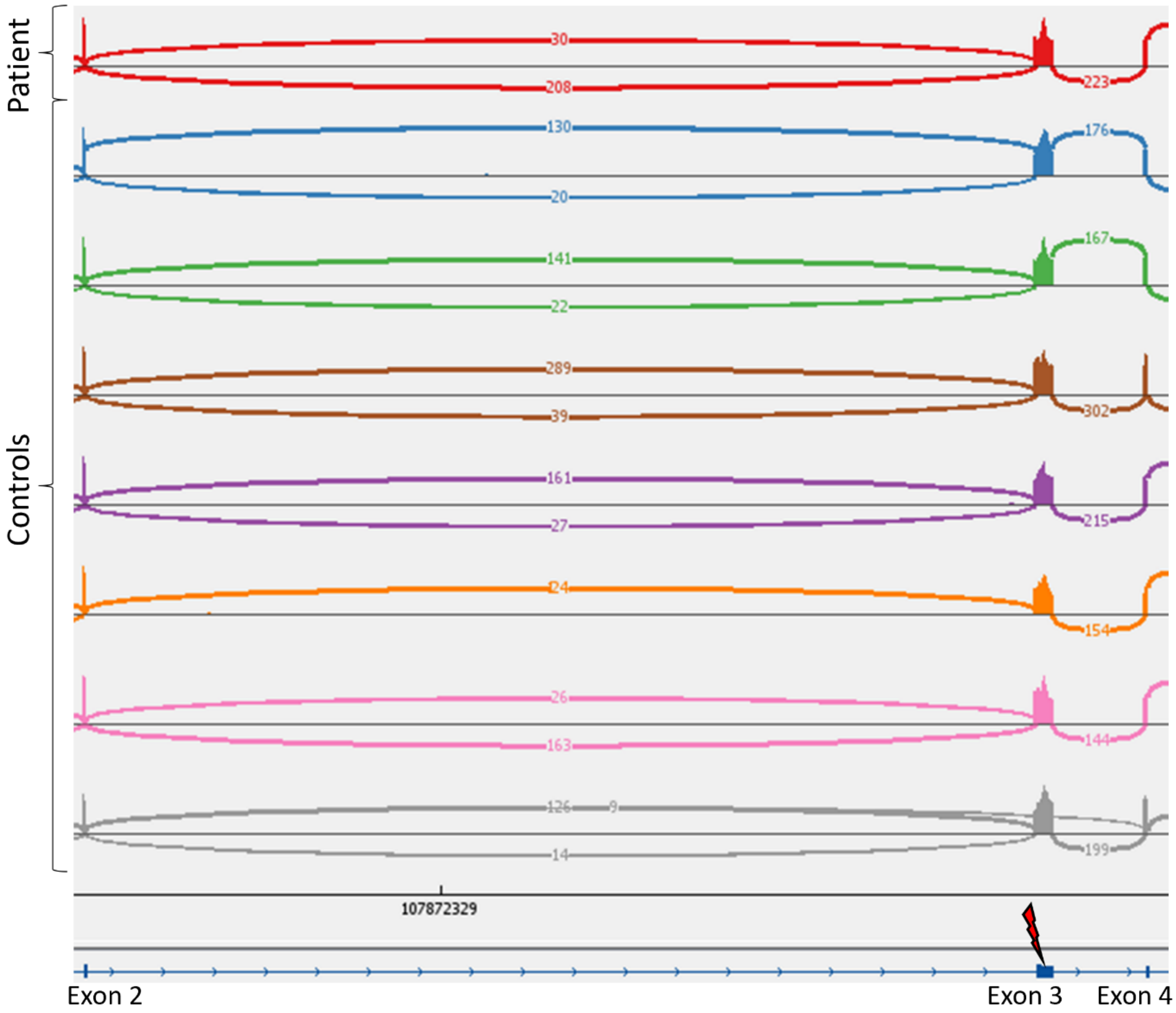

Supplement figure S6. Alternative splicing analysis between the patient with the p.Met64Arg variant and controls. No splicing differences were seen between the patient and control samples. The red lightning bolt indicates the exon with the p.Met64Arg variant. The reference transcript used was NM_001375905.

STAR(V2.7.10a). Essential parameter settings different from the default.

Parameter	Value
outFilterType	BySJout
outFilterMultimapNmax	20
alignSJoverhangMin	8
alignSJDBoverhangMin	1
outFilterMismatchNmax	999
outFilterMismatchNoverReadLmax	0.04
alignIntronMin	20
alignIntronMax	1000000
alignMatesGapMax	1000000
outSAMunmapped	Within
outSAMattributes	All
sjdbScore	1
outMultimapperOrder	Random
limitOutSJcollapsed	2000000
twopassMode	Basic

Primer sequences for every target and reference gene used in the study.

Gene	Direction	Sequence (5´-3´)	Class
ELFN1	Fw	GGCAGCTACATGGAGGTTCGAA	Target gene
	Rv	TCTGGTTGACCTTGTCCACCTC	
MOXD1	Fw	GCACTTTGGAGTGCCTGGAAGA	Target gene
	Rv	AATGACGCAGCCTGATGCCTCT	
CUL7	Fw	CCGCAAACTCATCACCAACATCC	Target gene
	Rv	GGCACAGGTATCTGAGGAACAC	
ACTC1	Fw	TCTGGCTCCTAGCACCATGAAG	Target gene
	Rv	GCCTCATCGTACTCTTGCTTGC	
JCAD	Fw	CTTCTGCTTCCAGAACCTCGGA	Target gene
	Rv	TGGCTTCTTGCCGTCCTCTTCT	
NACC2	Fw	CTCATCAGCCAGATCGGATACC	Target gene
	Rv	ACACAGGTGGCAGTTCATCAGC	
TBP	Fw	GAGCTGTGATGTGAAGTTTCC	Reference gene
	Rv	TCTGGGTTTGATCATTCTGTAG	

Liquid-liquid extraction (LLE) method

LLE for serum and cell samples proceeded as follows: for the serum samples, 100µl of the sample and 1ml of water were transferred to a glass tube. For the cell samples, 1 ml of water was added for sonication at a frequency of 37, power of 60 with no temperature for 1 minute followed by vortexing for 2 minutes in three cycles. The resulting mixture was then transferred to glass tubes. Next, 1 ml of methanol along with 100µL of a labelled internal standard mixture (prepared following the instructions in the SCIEX lipidyzer manual) was added to both the serum and the cell samples. The mixture was allowed to equilibrate with

the samples. After that, 3.5 mL of ethyl acetate was added, and the sample tubes were placed on a rotator shaker for 15 minutes at 30 rpm. This was followed by centrifugation at 3000 rpm for 10 minutes. After centrifugation, the upper layer of ethyl acetate was collected and dried under nitrogen. Dried samples were reconstituted with 250µL of mobile phase (Dichloromethane: Methanol (50:50) containing 10mM ammonium acetate) for injection.

Lipid separation and quantification were done using SCIEX LIPIDYZER platform utilizing an ABSCIEX 5500 QTRAP mass spectrometer (SCIEX, Framingham, MA, USA) with SelexIon (differential ion mobility) DMS technology. The analysis involved direct infusion of 50µL of extracted samples with the mobile phase at a flow rate of 70µL/min. Two acquisition methods, with and without SelexION® technology, were used covering 13 lipid classes using a flow injection analysis (FIA). Internal standard kits containing 50 labeled internal standards across 13 lipid classes were purchased from AB Sciex (Framingham, MA, USA) and used for lipid quantification. The lipid molecular species were measured using MRM strategy in both positive and negative polarities. Positive ion mode was used for detection of lipid classes – Sphingomyelins (SM)/Diacylglycerols (DAG)/Cholesterol Esters (CE)/ Ceramides (CER)/Triacylglycerols (TAG) and negative ion mode was used for detection of lipid classes Lysophosphatidylethanolamine(LPE)/Lysophosphatidylcholine(LPC)/Phosphatidylcholines(PC)/Phosphatidylethanolamine (PE)/Free Fatty Acids (FFA). Lipidomics Workflow Manager software (SCIEX, Framingham, MA, USA) was used for samples acquisition and for automated data-processing, signal detection and lipids species concentrations calculations.

QC samples, in-house serum samples from Institute for Molecular Medicine Finland (FIMM), were interspersed within the run such that after every 10th customer samples there was one QC sample to check integrity of the run in terms of analysis. System suitability test was performed before submitting the samples and experiment was passed. Also, no abnormalities were observed in sample quality, or lipid extraction, or mass spectrometry run, hence, overall results were acceptable.
